# 8*H*-Chromeno[2′,3′:4,5]imidazo[2,1-*a*]isoquinoline

**DOI:** 10.1107/S1600536810006744

**Published:** 2010-02-27

**Authors:** Saifidin Safarov, Leonid G. Voskressensky, Oksana V. Bizhko, Larisa N. Kulikova, Victor N. Khrustalev

**Affiliations:** a V. I. Nikitin Institute of Chemistry, Ayni St. 299/2, Dushanbe 734063, Tajikistan; b Organic Chemistry Department, Russian Peoples Friendship University, Miklukho-Maklai St 6, Moscow 117198, Russian Federation; c A. N. Nesmeyanov Institute of Organoelement Compounds, Russian Academy of Sciences, Vavilov St 28, B-334, Moscow 119991, Russian Federation

## Abstract

The title compound, C_18_H_12_N_2_O, comprises two aromatic fragments, *viz.*, imidazo[2,1-*a*]isoquinoline and benzene, linked by oxygen and methyl­ene bridges. Despite the absence of a common conjugative system within the mol­ecule, it adopts an essentially planar conformation with an r.m.s. deviation of 0. 036 Å. In the crystal, due to this structure, mol­ecules form stacks along the *b* axis by π⋯π stacking inter­actions, with shortest C⋯C distances in the range 3.340 (4)–3.510 (4) Å. The mol­ecules are bound by inter­molecular C—H⋯O inter­actions within the stacks and C—H⋯π inter­actions between the stacks.

## Related literature

For background to cascade reactions, see: Bunce (1995 ▶); Tietze (1996 ▶); Parsons *et al.* (1996 ▶); Nicolaou *et al.* (2003 ▶, 2006 ▶); Wasilke *et al.* (2005 ▶); Pellissier (2006*a* ▶,*b* ▶); Parenty & Cronin (2008 ▶). For related compounds, see: Yadav *et al.* (2007 ▶); Kianmehr *et al.* (2009 ▶); Surpur *et al.* (2009 ▶).
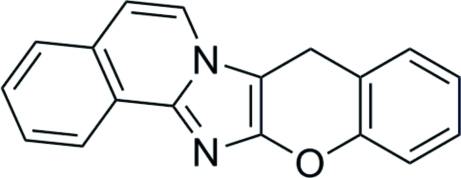


## Experimental

### 

#### Crystal data

C_18_H_12_N_2_O
*M* *_r_* = 272.30
Monoclinic, 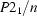

*a* = 11.9717 (15) Å
*b* = 6.0580 (8) Å
*c* = 17.948 (2) Å
β = 102.682 (3)°
*V* = 1269.9 (3) Å^3^
*Z* = 4
Mo *K*α radiation
μ = 0.09 mm^−1^
*T* = 100 K
0.40 × 0.12 × 0.02 mm

#### Data collection

Bruker APEXII CCD diffractometer
Absorption correction: multi-scan (*SADABS*; Sheldrick, 2003 ▶) *T*_min_ = 0.965, *T*_max_ = 0.998
12413 measured reflections
2734 independent reflections
1821 reflections with *I* > 2σ(*I*)
*R*_int_ = 0.056

#### Refinement

*R*[*F* ^2^ > 2σ(*F* ^2^)] = 0.066
*wR*(*F* ^2^) = 0.182
*S* = 1.00
2734 reflections
190 parameters
H-atom parameters constrained
Δρ_max_ = 0.45 e Å^−3^
Δρ_min_ = −0.23 e Å^−3^

### 

Data collection: *APEX2* (Bruker, 2005 ▶); cell refinement: *SAINT-Plus* (Bruker, 2001 ▶); data reduction: *SAINT-Plus*; program(s) used to solve structure: *SHELXS97* (Sheldrick, 2008 ▶); program(s) used to refine structure: *SHELXL97* (Sheldrick, 2008 ▶); molecular graphics: *SHELXTL* (Sheldrick, 2008 ▶); software used to prepare material for publication: *SHELXTL*.

## Supplementary Material

Crystal structure: contains datablocks global, I. DOI: 10.1107/S1600536810006744/rk2193sup1.cif

Structure factors: contains datablocks I. DOI: 10.1107/S1600536810006744/rk2193Isup2.hkl

Additional supplementary materials:  crystallographic information; 3D view; checkCIF report

## Figures and Tables

**Table 1:** Hydrogen-bond geometry (Å, °) *Cg*2 is the centroid of the O13,C12*A*,C8*A*,C8,C7*A*,C13*A* ring.

*D*—H⋯*A*	*D*—H	H⋯*A*	*D*⋯*A*	*D*—H⋯*A*
C8—H8*A*⋯O13^i^	0.99	2.71	3.637 (4)	157
C8—H8*B*⋯*Cg*^ii^	0.99	2.63	3.547 (3)	154

Symmetry codes: (i) 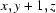; (ii) 
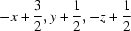
.

**Fig. 1.**
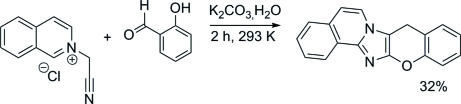
Synthesis of compound I.

**Fig. 2.**
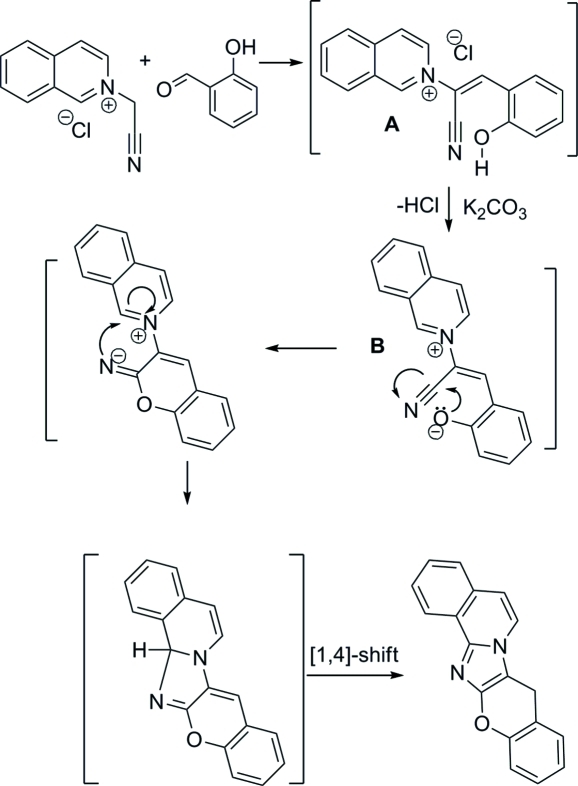
The plausible formation mechanism of I.

**Fig. 3.**
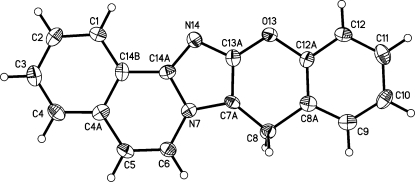
Molecular structure of Iwith the atom numbering scheme. Displacement ellipsoids are shown atthe 50% probability level. H atoms are presented as a small spheres of arbitrary radius.

**Fig. 4.**
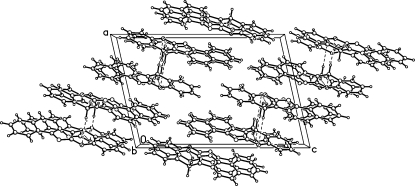
Crystal packing of I viewed down the b axis. Dashed lines indicate the C—H···O and C—H···π interactions.

## supplementary crystallographic information

### Comment

Cascade reactions have emerged as powerful tools to allow rapidly increasing molecular complexity (Tietze, 1996; Parsons *et al.*, 1996; Wasilke *et al.*, 2005). These processes avoid the excessive handling and isolation of synthetic intermediates generating less waste and thus contribute towards "Green Chemistry". Cascade reactions, in which multiple reactions are combined into one synthetic operation, have been reported extensively in the literature and have already become "state–of–the–art" in synthetic organic chemistry (Bunce, 1995; Nicolaou *et al.*, 2003, 2006; Pellissier, 2006*a*, 2006*b*; Parenty & Cronin, 2008).

The title compound I, C_18_H_12_N_2_O, is the product of a novel cascade reaction (Fig. 1) (Yadav *et al.*, 2007; Kianmehr *et al.*, 2009; Surpur *et al.*, 2009) starting with the Kroehnke condensation of salicylic aldehyde and isoquinolinium salt to afford the styryl derivative A, which forms zwitterion B upon thermally–induced cleavage of acetyl chloride. Then zwitterion B undergoes two consecutive nucleophilic cyclizations followed by [1,4]–proton shift to give the pentacycle I (Fig. 2). The single crystals of I suitable for X–ray diffraction analysis were obtained by slow crystallization from ethyl acetate solution.

Compound I comprises two aromatic fragments - imidazo[2,1–*a*]isoquinoline and benzene linked by the oxygen and methylene bridges (Fig. 3). Despite the absence of common conjugative system within the molecule, it adopts practically planar conformation, with the r.m.s. deviation of 0.036Å. In the crystal, due to this structure, molecules form stacks along the *b* axis by the stacking interactions [C1···C7A^i^ = 3.340 (4)Å, C2···C8^i^ = 3.510Å, C2···C8A^i^ = 3.451 (4)Å, C3···C12A^i^ = 3.394 (4)Å, C13A···C14B^i^ = 3.496 (4)Å and C14A···C14A^i^ = 3.426 (4)Å] (Fig. 4). The molecules are also bound by the C8—H8A···O13^ii^ [H···O = 2.71Å, C—H···O 157°] interactions within the stacks and the C8—H8B···π (C12A^iii^—O13^iii^—C13A^iii^) [H···C12A = 2.94Å, H···O13 = 2.80Å and H···C13A 2.81Å, C—H···O 175°] interactions between the stacks. Symmetry codes: (i) 1-*x*, 1-*y*, *z*; (ii) *x*, 1+*y*, *z*; (iii) 1.5-*x*, 0.5+*y*, 0.5-*z*.

### Experimental

A water solution of K_2_CO_3 _(0.4 g in 1 ml of H_2_O) was added to a solution of freshly distilled salicylic aldehyde (0.18 g, 1.47 mmol) and 2–(cyanomethyl)isoquinolinium chloride (0.30 g, 1.47 mmol) in H_2_O (5 ml). The resulting mixture was stirred for 3 hours at 293 K. The precipitate formed was filtered–off and recrystallized from ethyl acetate / hexane mixture to give product I as colourless needles. Yield is 32%. M.p. = 444 K. Found (%): C 79.13, H4.58, N 10.53. Calcd. for C_18_H_12_N_2_O (%): C79.39, H 4.44, N 10.29. ^1^H NMR (400 MHz, CDCl_3_): δ = 4.25 (s, 2H, CH_2_), 6.98 (dd, 1H, H12, *J*_11,12_ = 8.1, *J*_10,12_ = 1.2), 7.01 (d, 1H, H5, *J*_5,6_ = 7.5), 7.07–7.12 (m, 2H, H9+H10), 7.16 (dd, 1H, H11, *J*_11,12_ = 8.1, *J*_9,11_ = 1.2), 7.40–7.45 (m, 1H, H3), 7.48–7.53 (m, 1H, H2), 7.55 (d, 1H, H4, *J*_3,4_ = 7.5), 7.58 (d, 1H, H6, *J*_5,6_ = 7.5), 8.47(d, 1H, H1, *J*_1,2_ = 8.1). ^13^C NMR (100 MHz, CDCl_3_): δ = 23.2 (CH_2_), 112.9 (CH), 117.8 (C_q_), 118.1 (C_q_), 118.3 (CH), 120.3 (CH), 123.1 (CH), 123.2 (C_q_), 123.5 (CH), 127.1 (CH), 127.8 (CH), 128.2 (2xCH), 129.1 (C_q_), 130.3 (CH), 138.0 (C_q_), 152.0 (C_q_), 161.1 (C_q_). Mass spectrum (EI MS), m/z (I_r_, %): 272 (70) [*M*^+.^], 136 (11), 128 (10).

### Refinement

The hydrogen atoms were placed in calculated positions with C—H = 0.95–0.99Å and refined in the riding model with fixed isotropic displacement parameters [*U*_iso_(H) = 1.2*U*_eq_(C)].

### Crystal data

**Table:**

C_18_H_12_N_2_O	*F*(000) = 568
*M**_r_* = 272.30	*D*_x_ = 1.424 Mg m^−^^3^
Monoclinic, *P*2_1_/*n*	Mo *K*α radiation, λ = 0.71073 Å
Hall symbol: -P 2yn	Cell parameters from 1700 reflections
*a* = 11.9717 (15) Å	θ = 2.3–26.3°
*b* = 6.0580 (8) Å	µ = 0.09 mm^−^^1^
*c* = 17.948 (2) Å	*T* = 100 K
β = 102.682 (3)°	Needle, colourless
*V* = 1269.9 (3) Å^3^	0.40 × 0.12 × 0.02 mm
*Z* = 4	

### Data collection

**Table:**

Bruker APEXII CCD diffractometer	2734 independent reflections
Radiation source: fine–focus sealed tube	1821 reflections with *I* > 2σ(*I*)
graphite	*R*_int_ = 0.056
φ and ω scans	θ_max_ = 27.0°, θ_min_ = 1.9°
Absorption correction: multi-scan (*SADABS*; Sheldrick, 2003)	*h* = −15→15
*T*_min_ = 0.965, *T*_max_ = 0.998	*k* = −7→7
12413 measured reflections	*l* = −22→22

### Refinement

**Table:**

Refinement on *F*^2^	Primary atom site location: structure-invariant direct methods
Least-squares matrix: full	Secondary atom site location: difference Fourier map
*R*[*F*^2^ > 2σ(*F*^2^)] = 0.066	Hydrogen site location: inferred from neighbouring sites
*wR*(*F*^2^) = 0.182	H-atom parameters constrained
*S* = 1.00	*w* = 1/[σ^2^(*F*_o_^2^) + (0.077*P*)^2^ + 1.7*P*] where *P* = (*F*_o_^2^ + 2*F*_c_^2^)/3
2734 reflections	(Δ/σ)_max_ < 0.001
190 parameters	Δρ_max_ = 0.45 e Å^−^^3^
0 restraints	Δρ_min_ = −0.23 e Å^−^^3^

### Special details

**Table:**

Geometry. All s.u.'s (except the s.u. in the dihedral angle between two l.s. planes) are estimated using the full covariance matrix. The cell s.u.'s are taken into account individually in the estimation of s.u.'s in distances, angles and torsion angles; correlations between s.u.'s in cell parameters are only used when they are defined by crystal symmetry. An approximate (isotropic) treatment of cell s.u.'s is used for estimating s.u.'s involving l.s. planes.
Refinement. Refinement of *F*^2^ against ALL reflections. The weighted *R*–factor wR and goodness of fit *S* are based on *F*^2^, conventional *R*–factors *R* are based on *F*, with *F* set to zero for negative *F*^2^. The threshold expression of *F*^2^ > σ(*F*^2^) is used only for calculating *R*–factors(gt) etc. and is not relevant to the choice of reflections for refinement. *R*–factors based on *F*^2^ are statistically about twice as large as those based on *F*, and *R*–factors based on ALL data will be even larger.

### Fractional atomic coordinates and isotropic or equivalent isotropic displacement parameters (Å^2^)

**Table:**

	*x*	*y*	*z*	*U*_iso_*/*U*_eq_	
C1	0.6165 (2)	0.1766 (5)	−0.07577 (16)	0.0290 (6)	
H1	0.5719	0.0665	−0.0583	0.035*	
C2	0.6436 (2)	0.1563 (5)	−0.14599 (17)	0.0349 (7)	
H2	0.6185	0.0302	−0.1766	0.042*	
C3	0.7068 (2)	0.3167 (6)	−0.17271 (17)	0.0372 (7)	
H3	0.7237	0.3008	−0.2217	0.045*	
C4	0.7447 (2)	0.4957 (5)	−0.12988 (17)	0.0349 (7)	
H4	0.7885	0.6033	−0.1494	0.042*	
C4A	0.7213 (2)	0.5276 (5)	−0.05755 (16)	0.0309 (6)	
C5	0.7591 (2)	0.7182 (5)	−0.01180 (16)	0.0332 (7)	
H5	0.8052	0.8253	−0.0295	0.040*	
C6	0.7306 (2)	0.7478 (5)	0.05559 (16)	0.0310 (6)	
H6	0.7538	0.8777	0.0845	0.037*	
N7	0.66701 (19)	0.5884 (4)	0.08314 (13)	0.0269 (5)	
C7A	0.6332 (2)	0.5823 (4)	0.15238 (15)	0.0251 (6)	
C8	0.6498 (2)	0.7432 (5)	0.21381 (16)	0.0326 (7)	
H8A	0.6141	0.8858	0.1950	0.039*	
H8B	0.7325	0.7676	0.2349	0.039*	
C8A	0.5921 (2)	0.6458 (5)	0.27520 (16)	0.0301 (6)	
C9	0.5902 (2)	0.7644 (5)	0.34032 (18)	0.0347 (7)	
H9	0.6261	0.9050	0.3466	0.042*	
C10	0.5385 (3)	0.6886 (5)	0.39654 (18)	0.0382 (7)	
H10	0.5390	0.7752	0.4407	0.046*	
C11	0.4851 (2)	0.4809 (6)	0.38774 (18)	0.0387 (8)	
H11	0.4483	0.4261	0.4258	0.046*	
C12	0.4864 (2)	0.3558 (5)	0.32275 (16)	0.0304 (6)	
H12	0.4516	0.2141	0.3163	0.037*	
C12A	0.5393 (2)	0.4417 (5)	0.26757 (15)	0.0264 (6)	
O13	0.53167 (16)	0.2981 (3)	0.20489 (11)	0.0319 (5)	
C13A	0.5786 (2)	0.3795 (5)	0.14856 (15)	0.0297 (6)	
N14	0.57698 (19)	0.2676 (4)	0.08385 (13)	0.0297 (5)	
C14A	0.6311 (2)	0.3960 (5)	0.04379 (16)	0.0291 (6)	
C14B	0.6554 (2)	0.3618 (5)	−0.02983 (14)	0.0278 (6)	

### Atomic displacement parameters (Å^2^)

**Table:**

	*U*^11^	*U*^22^	*U*^33^	*U*^12^	*U*^13^	*U*^23^
C1	0.0230 (13)	0.0287 (15)	0.0340 (15)	−0.0002 (11)	0.0032 (11)	0.0065 (12)
C2	0.0304 (15)	0.0366 (16)	0.0339 (16)	0.0086 (13)	−0.0012 (12)	−0.0084 (13)
C3	0.0285 (15)	0.056 (2)	0.0270 (15)	0.0063 (14)	0.0051 (12)	0.0020 (14)
C4	0.0300 (15)	0.0386 (17)	0.0360 (17)	0.0009 (13)	0.0072 (12)	0.0117 (13)
C4A	0.0256 (13)	0.0281 (15)	0.0354 (16)	0.0053 (11)	−0.0012 (12)	−0.0019 (12)
C5	0.0312 (15)	0.0325 (15)	0.0360 (16)	−0.0054 (12)	0.0080 (12)	0.0073 (13)
C6	0.0333 (15)	0.0264 (14)	0.0335 (15)	0.0002 (12)	0.0078 (12)	0.0053 (12)
N7	0.0261 (11)	0.0246 (12)	0.0282 (12)	0.0025 (9)	0.0021 (9)	0.0000 (10)
C7A	0.0187 (12)	0.0265 (14)	0.0297 (14)	−0.0019 (10)	0.0045 (10)	0.0056 (11)
C8	0.0258 (14)	0.0372 (16)	0.0350 (16)	−0.0040 (12)	0.0072 (12)	−0.0058 (13)
C8A	0.0209 (13)	0.0325 (15)	0.0350 (15)	0.0021 (11)	0.0020 (11)	0.0023 (13)
C9	0.0278 (14)	0.0313 (15)	0.0440 (17)	0.0019 (12)	0.0055 (12)	0.0008 (13)
C10	0.0365 (16)	0.0407 (18)	0.0362 (16)	0.0091 (14)	0.0054 (13)	−0.0132 (14)
C11	0.0304 (15)	0.051 (2)	0.0388 (17)	0.0089 (14)	0.0175 (13)	0.0082 (15)
C12	0.0239 (13)	0.0275 (14)	0.0397 (16)	0.0001 (11)	0.0067 (12)	0.0026 (13)
C12A	0.0214 (12)	0.0294 (14)	0.0283 (14)	0.0068 (11)	0.0055 (11)	−0.0016 (11)
O13	0.0344 (11)	0.0285 (10)	0.0352 (11)	−0.0058 (8)	0.0130 (9)	−0.0022 (9)
C13A	0.0256 (14)	0.0345 (15)	0.0290 (14)	0.0048 (12)	0.0059 (11)	0.0012 (12)
N14	0.0254 (11)	0.0327 (13)	0.0314 (13)	0.0016 (10)	0.0070 (9)	0.0015 (10)
C14A	0.0252 (13)	0.0257 (14)	0.0347 (15)	0.0004 (11)	0.0029 (11)	−0.0018 (12)
C14B	0.0227 (13)	0.0376 (16)	0.0214 (13)	0.0105 (11)	0.0014 (10)	0.0021 (12)

### Geometric parameters (Å, °)

**Table:**

C1—C2	1.374 (4)	C8—C8A	1.540 (4)
C1—C14B	1.410 (4)	C8—H8A	0.9900
C1—H1	0.9500	C8—H8B	0.9900
C2—C3	1.380 (5)	C8A—C9	1.377 (4)
C2—H2	0.9500	C8A—C12A	1.382 (4)
C3—C4	1.349 (5)	C9—C10	1.373 (4)
C3—H3	0.9500	C9—H9	0.9500
C4—C4A	1.400 (4)	C10—C11	1.405 (5)
C4—H4	0.9500	C10—H10	0.9500
C4A—C5	1.432 (4)	C11—C12	1.394 (4)
C4A—C14B	1.432 (4)	C11—H11	0.9500
C5—C6	1.339 (4)	C12—C12A	1.389 (4)
C5—H5	0.9500	C12—H12	0.9500
C6—N7	1.386 (4)	C12A—O13	1.409 (3)
C6—H6	0.9500	O13—C13A	1.353 (3)
N7—C14A	1.382 (4)	C13A—N14	1.341 (4)
N7—C7A	1.390 (4)	N14—C14A	1.321 (4)
C7A—C13A	1.386 (4)	C14A—C14B	1.429 (4)
C7A—C8	1.452 (4)		
			
C2—C1—C14B	119.6 (3)	C8A—C8—H8B	110.5
C2—C1—H1	120.2	H8A—C8—H8B	108.7
C14B—C1—H1	120.2	C9—C8A—C12A	117.3 (3)
C1—C2—C3	120.9 (3)	C9—C8A—C8	120.1 (3)
C1—C2—H2	119.6	C12A—C8A—C8	122.6 (3)
C3—C2—H2	119.6	C10—C9—C8A	122.9 (3)
C4—C3—C2	120.6 (3)	C10—C9—H9	118.5
C4—C3—H3	119.7	C8A—C9—H9	118.5
C2—C3—H3	119.7	C9—C10—C11	119.0 (3)
C3—C4—C4A	121.9 (3)	C9—C10—H10	120.5
C3—C4—H4	119.0	C11—C10—H10	120.5
C4A—C4—H4	119.0	C12—C11—C10	119.5 (3)
C4—C4A—C5	122.7 (3)	C12—C11—H11	120.2
C4—C4A—C14B	117.6 (3)	C10—C11—H11	120.2
C5—C4A—C14B	119.7 (3)	C12A—C12—C11	118.9 (3)
C6—C5—C4A	121.0 (3)	C12A—C12—H12	120.5
C6—C5—H5	119.5	C11—C12—H12	120.5
C4A—C5—H5	119.5	C8A—C12A—C12	122.4 (3)
C5—C6—N7	119.9 (3)	C8A—C12A—O13	125.4 (2)
C5—C6—H6	120.1	C12—C12A—O13	112.2 (2)
N7—C6—H6	120.1	C13A—O13—C12A	114.0 (2)
C14A—N7—C6	122.6 (2)	N14—C13A—O13	122.2 (3)
C14A—N7—C7A	108.4 (2)	N14—C13A—C7A	114.1 (2)
C6—N7—C7A	129.0 (2)	O13—C13A—C7A	123.7 (2)
C13A—C7A—N7	101.9 (2)	C14A—N14—C13A	104.9 (2)
C13A—C7A—C8	128.1 (2)	N14—C14A—N7	110.7 (2)
N7—C7A—C8	130.0 (2)	N14—C14A—C14B	129.9 (3)
C7A—C8—C8A	106.1 (2)	N7—C14A—C14B	119.4 (3)
C7A—C8—H8A	110.5	C1—C14B—C14A	123.3 (3)
C8A—C8—H8A	110.5	C1—C14B—C4A	119.4 (2)
C7A—C8—H8B	110.5	C14A—C14B—C4A	117.3 (3)
			
C14B—C1—C2—C3	−1.1 (4)	C11—C12—C12A—O13	−178.9 (2)
C1—C2—C3—C4	0.9 (4)	C8A—C12A—O13—C13A	−2.1 (4)
C2—C3—C4—C4A	−0.5 (4)	C12—C12A—O13—C13A	177.7 (2)
C3—C4—C4A—C5	−178.9 (3)	C12A—O13—C13A—N14	−178.5 (2)
C3—C4—C4A—C14B	0.2 (4)	C12A—O13—C13A—C7A	2.1 (4)
C4—C4A—C5—C6	176.9 (3)	N7—C7A—C13A—N14	−0.3 (3)
C14B—C4A—C5—C6	−2.3 (4)	C8—C7A—C13A—N14	180.0 (3)
C4A—C5—C6—N7	2.4 (4)	N7—C7A—C13A—O13	179.1 (2)
C5—C6—N7—C14A	0.2 (4)	C8—C7A—C13A—O13	−0.6 (4)
C5—C6—N7—C7A	176.8 (3)	O13—C13A—N14—C14A	−179.3 (2)
C14A—N7—C7A—C13A	0.3 (3)	C7A—C13A—N14—C14A	0.1 (3)
C6—N7—C7A—C13A	−176.7 (3)	C13A—N14—C14A—N7	0.1 (3)
C14A—N7—C7A—C8	−179.9 (3)	C13A—N14—C14A—C14B	179.9 (3)
C6—N7—C7A—C8	3.1 (5)	C6—N7—C14A—N14	177.0 (2)
C13A—C7A—C8—C8A	−1.0 (4)	C7A—N7—C14A—N14	−0.3 (3)
N7—C7A—C8—C8A	179.3 (2)	C6—N7—C14A—C14B	−2.8 (4)
C7A—C8—C8A—C9	−177.9 (2)	C7A—N7—C14A—C14B	179.9 (2)
C7A—C8—C8A—C12A	1.0 (4)	C2—C1—C14B—C14A	179.9 (3)
C12A—C8A—C9—C10	−0.1 (4)	C2—C1—C14B—C4A	0.8 (4)
C8—C8A—C9—C10	178.9 (3)	N14—C14A—C14B—C1	3.9 (4)
C8A—C9—C10—C11	0.0 (4)	N7—C14A—C14B—C1	−176.3 (2)
C9—C10—C11—C12	0.6 (4)	N14—C14A—C14B—C4A	−176.9 (3)
C10—C11—C12—C12A	−1.0 (4)	N7—C14A—C14B—C4A	2.8 (4)
C9—C8A—C12A—C12	−0.4 (4)	C4—C4A—C14B—C1	−0.4 (4)
C8—C8A—C12A—C12	−179.3 (2)	C5—C4A—C14B—C1	178.8 (2)
C9—C8A—C12A—O13	179.4 (2)	C4—C4A—C14B—C14A	−179.5 (2)
C8—C8A—C12A—O13	0.5 (4)	C5—C4A—C14B—C14A	−0.4 (4)
C11—C12—C12A—C8A	0.9 (4)		

### Hydrogen-bond geometry (Å, °)

**Table:**

Cg2 is the centroid of the O13,C12A,C8A,C8,C7A,C13A ring.

**Table:**

*D*—H···*A*	*D*—H	H···*A*	*D*···*A*	*D*—H···*A*
C8—H8A···O13^i^	0.99	2.71	3.637 (4)	157
C8—H8B···Cg^ii^	0.99	2.63	3.547 (3)	154

Symmetry codes: (i) *x*, *y*+1, *z*; (ii) −*x*+3/2, *y*+1/2, −*z*+1/2.

## References

[bb1] Bruker (2001). *SAINT-Plus* Bruker AXS Inc., Madison, Wisconsin, USA.

[bb2] Bruker (2005). *APEX2* Bruker AXS Inc., Madison, Wisconsin, USA.

[bb3] Bunce, R. A. (1995). *Tetrahedron*, **51**, 13103–13159.

[bb4] Kianmehr, E., Faramarzi, R. & Estiri, H. (2009). *Heterocycles*, **78**, 415–423.

[bb5] Nicolaou, K. C., Edmonds, D. J. & Bulger, P. G. (2006). *Angew. Chem. Int. Ed.* **45**, 7134–7186. 10.1002/anie.200601872 17075967

[bb6] Nicolaou, K. C., Montagnon, T. & Snyder, S. A. (2003). *Chem. Commun.* pp. 551–564. 10.1039/b209440c 12669826

[bb7] Parenty, A. D. C. & Cronin, L. (2008). *Synthesis*, pp. 1479–1485.

[bb8] Parsons, P. J., Penkett, C. S. & Shell, A. J. (1996). *Chem. Rev.* **96**, 195–206. 10.1021/cr950023+ 11848750

[bb9] Pellissier, H. (2006*a*). *Tetrahedron*, **62**, 1619–1665.

[bb10] Pellissier, H. (2006*b*). *Tetrahedron*, **62**, 2143–2173.

[bb11] Sheldrick, G. M. (2003). *SADABS* University of Göttingen, Germany.

[bb12] Sheldrick, G. M. (2008). *Acta Cryst.* A**64**, 112–122. 10.1107/S0108767307043930 18156677

[bb13] Surpur, M. P., Kshirsagar, S. & Samant, S. D. (2009). *Tetrahedron Lett.* **50**, 719–722.

[bb14] Tietze, L. F. (1996). *Chem. Rev.* **96**, 115–136. 10.1021/cr950027e 11848746

[bb15] Wasilke, J.–C., Obrey, S. J., Baker, R. T. & Bazan, G. C. (2005). *Chem. Rev.* **105**, 1001–1020. 10.1021/cr020018n 15755083

[bb16] Yadav, J. S., Subba Reddy, B. V., Gupta, M. K., Prathap, I. & Pandey, S. K. (2007). *Catal. Commun.* **8**, 2208–2211.

